# The State-Before-Event Inference Emerges Across Tenses

**DOI:** 10.1162/opmi_a_00207

**Published:** 2025-05-23

**Authors:** Elena Marx, Natalia Jardón, Eva Wittenberg

**Affiliations:** Cognitive Science Department, Central European University, Vienna, Austria

**Keywords:** relative clause constructions, dynamicity, tense semantics, modality, temporal anchoring, Figure-Ground, complex event construal

## Abstract

In language, comprehenders often need to infer the temporal order of events to construct a mental model of a complex situation. Dynamicity differences are a key predictor of these inferences: Non-dynamic states are reliably inferred to precede dynamic events. In two studies, we test two theoretical explanations for this phenomenon through temporal order judgments for past-under-past and future-under-future relative clauses in English: According to a tense-mediated account of temporal anchoring, people rely on the conceptual distinction between a more salient reference time—often a dynamic event—and a less salient anchored situation—often a static state. The temporal relationship between the two is determined at the linguistic level by tense meaning: For the past tense, the relationship should be one of anteriority, and for the future tense, it should be one of posteriority. However, the future tense has often been placed closer to modals than to tenses, relegating the question of temporal order to other mechanisms. Alternatively, from a purely cognitive perspective, salience differences between states and events are sufficient to infer temporal order, with states acting as temporal backgrounds for more salient events, regardless of tense. Our results support such a cognitive mechanism: In both experiments, states are backgrounded relative to events. Differences between the experiments furthermore support modal accounts of the semantics of the future.

## INTRODUCTION

The way in which comprehenders arrange situations in time is a crucial aspect of language comprehension, shaping the mental representation of any described complex scene (Radvansky et al., 1998; Zwaan et al., 1995): For example, hearing that the monkey was sitting in the tree while the tourist took a picture of it describes a situation in which the tree is part of the photograph. Conversely, hearing that the tourist took a picture of the monkey which then went to sit in the tree leads to a different mental construal: one in which the tree is absent from the picture.

Different temporal orders can, of course, be explicitly encoded through linguistic devices such as temporal connectives (e.g., *before*, *after*), temporal adverbials (e.g., *then*, *previously*), or contrasting verb tenses (e.g., *sat* – *will sit*). However, the availability and use of such devices vary across languages (Bohnemeyer, 2009; Bybee & Dahl, 1989; Comrie, 1985; Haspelmath et al., 2005; Lin, 2003, 2006; Tonhauser, 2015). Even in languages with mandatory explicit markers, such as the English tense system, temporal relations between events are often left ambiguous. For example, in a sentence like *The tourist took a picture of the monkey that sat in the tree*, it is unclear whether the monkey sat in the tree before, during, or after the picture was taken. This raises the question: How do comprehenders infer temporal order when explicit markers are absent or ambiguous?

In previous work, we demonstrated that, in temporally ambiguous descriptions, people systematically rely on the dynamic properties of predicates to infer the temporal relations between situations (Marx et al., 2025; Marx & Wittenberg, 2022, 2024, 2025): static states are interpreted to happen before dynamic events (the ‘states-before-events inference’). Applying this states-before-events inference to the example above, comprehenders are likely to construe the monkey’s state of sitting in the tree as the temporal background to the event of the tourist taking a picture.

Crucially, our prior studies exclusively examined past tense. However, it remains unclear whether the states-before-events inference is specific to the simple past, or whether this pattern holds across tenses, specifically future tense, which intuitively relates to the utterance time as the past does—just mirrored into the opposite direction. In this paper, we therefore shift our focus to the future tense, to explore the generalizability of the states-before-events inference. As before, we use relative clause constructions, which link two propositions together without signaling a systematic causal, temporal, or conditional relationship (Thomson & Martinet, 1985).

Future contexts furthermore offer a way to evaluate two competing theoretical explanations for the states-before-events inference, examining the role of tense meaning more generally: a temporal anchoring account, which posits that temporal construal is mediated via tense meaning, and a conceptual Figure-Ground principle that operates independently of tense.

According to the temporal anchoring view (Carroll & von Stutterheim, 2010; Klein, 1994, 2009; von Stutterheim et al., 2003), people construe temporal relations from language by linking situations to one another, based on the salience differences between static states and dynamic events. Therefore, less salient states should be systematically anchored by more salient events, with tense determining the temporal relationship. This relationship should be one of anteriority in the case of the past tense, and arguably one of posteriority in the case of the future tense. Consequently, while states should be interpreted as preceding events in past-under-past contexts, the temporal order should reverse in future-under-future contexts: That is, states should be inferred to follow events in future tense.

This hypothesis, however, assumes that the future functions primarily as a tense (Demirdache & Uribe-Etxebarria, 1997; Giorgi & Pianesi, 1997; Kissine, 2008; Klein, 1994; Prior, 1957), encoding a futurative relationship between a described situation and the utterance time—an assumption that has been rebutted by alternative accounts conceptualizing the simple future as a marker of modality, similar to modal auxiliaries (Enç, 1996; Huddleston & Pullum, 2005; Palmer, 1986).

In contrast, a Figure-Ground explanation does not rely on tense at all. Instead, such accounts predict that states serve as the temporal background for events, based on the salience differences that are associated with these dynamic properties alone: Static states should consistently serve as temporal backgrounds to dynamic events in both past and future contexts.

In two experiments presented in this paper, we test these predictions, studying the cognitive mechanisms driving temporal construal in language comprehension in the absence of explicit temporal order cues. Additionally, we aim to contribute to the broader theoretical debate regarding the nature of the future—whether it functions primarily as a tense marker or as a modal marker—and investigate the role of tense meaning in temporal construal more broadly.

Our paper is structured as follows: We first review the tense-mediated temporal anchoring account in greater depth (Event Order Through Tense-Mediated Temporal Anchoring section), applying it both to the past and the future tense. We then discuss the conceptualization of the future as a marker of modality (Future as a Modal, Not a Tense section), which challenges the applicability of tense anchoring in future contexts. Following this, we turn to alternative strategies for temporal construal in future contexts (Alternative Accounts to Derive Temporal Order From Language section), focusing on a Figure-Ground principle that likewise accounts for previous findings in the simple past. Finally, we outline the predictions that form the basis of our study (The Current Studies and Predictions section).

### Event Order Through Tense-Mediated Temporal Anchoring

#### The Case of Simple Past.

How do comprehenders derive temporal order from temporally ambiguous linguistic descriptions? One account suggests a mechanism that links temporal construal closely to event structure and the meaning of tense: tense-mediated temporal anchoring. According to this view, tensed predicates encode complex temporal structures comprising three key parameters (Carroll & von Stutterheim, 2010; Klein, 1994, 2009; von Stutterheim et al., 2003): The first parameter, the situation time, refers to the entire time span of the situation described by the predicate. Thus, situation times always correspond to the non-finite predicate (e.g., *taking a picture*, *sitting*) and rely on general event knowledge about duration and structure (e.g., taking a picture is punctual and culminates at the time the picture is shot, whereas sitting is durative and static over time).

Second, a topic time refers to a specific time about which the speaker makes an assertion, relative to the situation time. The relation between topic time and situation time is conveyed through aspect: For the simple past, which carries perfective aspect, the topic time corresponds to a part of the situation time, including its culmination point (i.e., for achievements and accomplishments) or post-state (i.e., for states and activities). However, topic time is always linked to the time the assertion is uttered, a relation established through tense: For the past tense, the topic time (and consequently parts of the situation time, including its culmination or post-state) is located at a time before the utterance time. For example, in *The tourist took a picture of the monkey*, the topic time denotes a period before the utterance time during which the event of taking the picture was completed.

Third, a temporal anchor makes reference to another temporal interval, linking it to the topic time. In isolation, like in a sentence such as *The tourist took a picture of the monkey*, the temporal anchor refers to the utterance time. In more complex event descriptions, however, the temporal anchor picks out another time interval mentioned in the description: In *The tourist took a picture of the monkey that sat in the tree*, either the time of the main clause situation (i.e., the tourist taking a picture) could serve as a temporal anchor for the relative clause situation (i.e., the monkey sitting in the tree) or the relationship could be reversed.

On a tense-anchoring account, two assumptions are important: First, temporal anchors are not chosen at random, but always correspond to the most salient temporal interval available. And given that the distinction between dynamic events and static states comes with a difference in saliency such that the former are cognitively more salient than the latter (Clewett et al., 2020; Kurby & Zacks, 2008; Zacks et al., 2007), eventive descriptions like *taking a picture*, which correspond to cognitively salient changes in the world, should serve as temporal anchors for static descriptions like *sitting*, which correspond to less salient, non-changing states.

**Figure 1.**
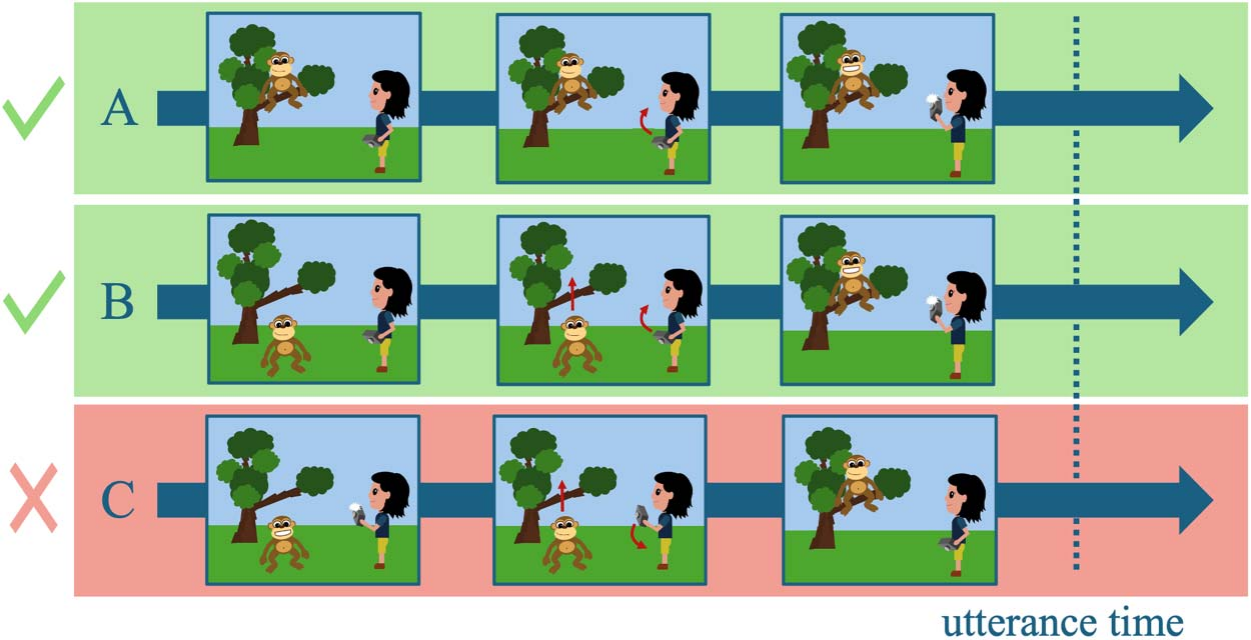
Possible temporal orders between two situations described by past-under-past relative clause constructions, according to a tense-mediated temporal anchoring account.

Second, because the temporal relation between situation time and topic time is tense mediated in this account, so should be the relation between the situation time and the temporal anchor: For simple past, the topic time spans parts of the situation time, including its culmination point or the post-state, which also imposes a logical constraint on its starting point: In *The tourist took a picture of the monkey that sat in the tree*, the anchored situation (i.e., monkey sitting in the tree) might begin before (Figure 1A), simultaneous (Figure 1B) but not after the time of the temporal anchor (i.e., tourist taking a picture, Figure 1C).

#### Temporal Anchoring and the Future Tense.

Throughout the past decades, future tense, and *will* in particular, has been analyzed by syntacticians and semanticists in (mainly) two ways: as a tense marker (Demirdache & Uribe-Etxebarria, 1997; Dowty, 1982; Giorgi & Pianesi, 1997; Kissine, 2008; Klein, 1994; Prior, 1957), or as a modal marker (Enç, 1996; Huddleston & Pullum, 2005; Palmer, 1986). The former regards the future as a tense, the mirror image of the past: According to this view, the future, like the past, denotes a temporal relation between the time of utterance and the topic time. This relation is represented in (1), for both tenses: the past locates the event in a time that precedes the utterance time (1a), whereas in the future this order is reversed (1b).

(1)  a. The tourist took a picture of the monkey.
      
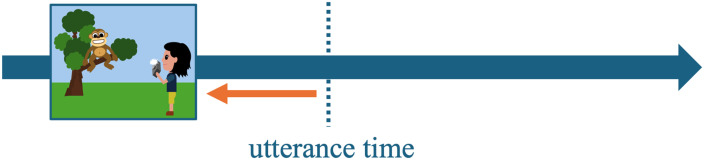
    b. The tourist will take a picture of the monkey.
      
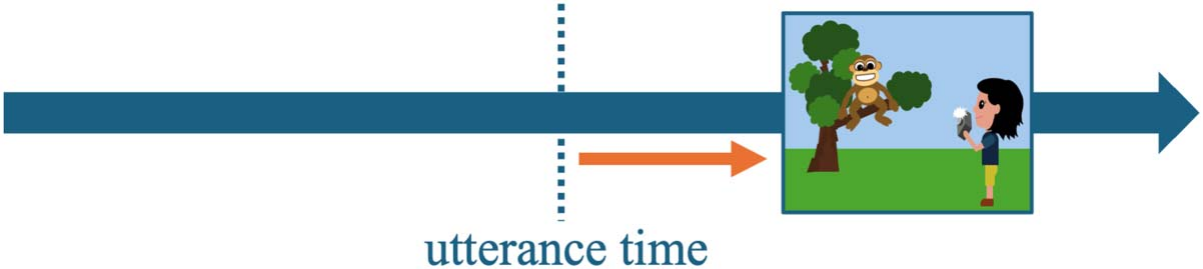

Deriving predictions about temporal order for future-under-future constructions is exceedingly difficult, since the literature either focuses on simple clauses (Demirdache & Uribe-Etxebarria, 1997; Dowty, 1982; Giorgi & Pianesi, 1997; Kissine, 2008; Klein, 1994; Ogihara, 1996; Prior, 1957) or complex utterances in which tenses are mixed (e.g., future-under-past, present-under-future, e.g., Abusch, 1998; Higginbotham, 2006; Ogihara, 1996). Therefore, we mainly base our predictions on logical extensions of the temporal anchoring account, with a caveat that more precise theoretical accounts are rare.^1^

If we apply the temporal anchoring approach to future-under-future constructions, the parameters of situation time, topic time, and temporal anchors as well as their interrelations are different from those in the simple past: For a sentence such as *The tourist will take a picture of the monkey that will sit in the tree*, the situation time corresponds to the non-finite part of the predicate (i.e., *take a picture, sit*), denoting both the duration and the internal structure of the situation described (i.e., dynamic, punctual *taking a picture* vs. static, durative *sitting*). This is similar to how situation time functions in the simple past.

The topic time, however, should span a different interval in future tense: With boundedness inherent to the simple future (as opposed to the future progressive, e.g., *will be sitting*), the topic time encompasses a time of the situation, including its culmination point or post-state. Crucially, the future tense locates the entire situation after the utterance time, including the situation’s starting point. Put simply, while the simple past restricts the culmination point to a time before the utterance, the future tense topic time signals that the situation has not yet started, but will unfold and eventually be completed at a later time, thus including both the starting and the ending points of the situation.

**Figure 2.**
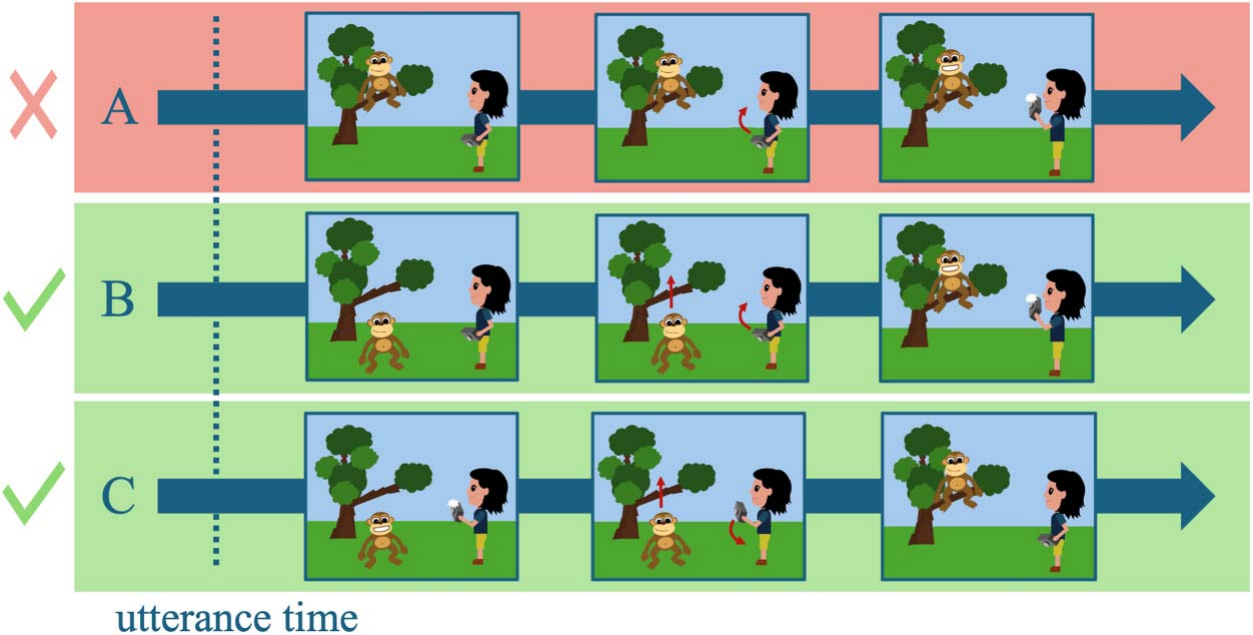
Possible temporal orders between two situations described by future-under-future relative clause constructions, derived from a tense-mediated temporal anchoring perspective.

This also has consequences for the temporal relationship between situation time and temporal anchor: In the example above, the temporal anchor is identified through saliency differences like in the simple past: The eventive description (i.e., *the tourist will take a picture*) establishes a salient reference time that anchors the stative description (i.e., *the monkey will sit in the tree*). However, since the topic time of the anchored situation now includes the entire situation time (i.e., including starting and ending point), the anchored situation (i.e., *monkey sitting in the tree*) cannot logically begin before the temporal anchor (i.e., *tourist taking a picture*, Figure 2A). Instead, it should be interpreted as co-occurring with (Figure 2B) or following the anchor event (Figure 2C).

### Future as a Modal, Not a Tense

In addition to the future-as-a-tense view, a second approach takes the future to be a modal (Enç, 1996; Huddleston & Pullum, 2005; Palmer, 1986), emphasizing its non-deictic nature. The idea is that, unlike the past, the future does not refer to a time, but only to potential occurrences of an event. Modal accounts of the future emphasize an asymmetry between future and past in terms of truth conditions, which are the standard test for well-formedness in formal semantics: Whereas it is easy to establish if a statement is true or false in the past, the same proof becomes problematic when the statement is about an event “in the future” (Brogaard, 2008; Heck, 2006). For instance, in evaluating the truth of the future-tense sentence in (2), the evaluation point lies in the future as well: Only after tomorrow, we will know whether this sentence was true.

(2)  We will submit this paper tomorrow.          (adapted from example (5), De Brabanter et al., 2014)

Under a modal view of the future, the abovementioned asymmetry follows naturally if the function of the future in a sentence is not to locate an event in a time that has not occurred yet, but rather if it serves a variety of (a)temporal purposes. In cases such as (3–5), adapted from Palmer (1974), Lapaire and Rotgé (1998), and Downing and Locke (2006), it is argued that *will* does not refer to a “future time”, but rather to some other meaning: dispositional (3); epistemic (4); habitual (5); or volitional (6).

(3)  Oil will float on water.
(4)  John will be at the opera by now.
(5)  In summer, Michael will(/would) always wear shorts.
(6)  The child will eat the cake (if he gets the chance).

The dispositional *will* in (3) states what is characteristic of oil, such that, according to its internal properties, and independently of time, it stands in a particular relationship to water; the epistemic *will* in (4) refers to how likely it is, according to what I know, that John is at the opera at that time. Again, it says nothing about John in a time following the present; the habitual (also dispositional) *will* in (5) takes the duration of the summer season as its temporal reference, and states that one can see Michael wearing shorts, or that Michael is a short-user, in that interval of the year; finally, the volitional *will* in (6) refers to the child’s intention or will (notice the word) to do something.

For those advocating a modal account of the future, the question becomes what kind of modality is at play: whether it is an instance of root modality, related to ability and volition, like *can* or *want* (Copley, 2002; Del Prete, 2014), or it is an instance of epistemic modality, along the lines of *must* (Giannakidou & Mari, 2012; Palmer, 1986). Independently of the specific modal flavor of the future, the modal approach predicts underspecification in temporal construals in the future, as compared to the past.^2^

Assuming that the simple future is not a true tense but a modal expression, this suggests that temporal order in future-tense contexts may rely more on alternative pragmatic cues than on tense-mediated temporal anchoring. The question is: What are alternative ways of deriving temporal order from language? Do dynamicity differences play a role in ordering events in time even when temporal reference is to the future rather than the past?

In the following chapter, we will outline three possible alternatives through which temporal order could be derived. One of the proposed mechanisms can also account for the patterns observed in past-under-past constructions by invoking a simpler mechanism based solely on event dynamicity: a Figure-Ground principle.

### Alternative Accounts to Derive Temporal Order From Language

#### Iconicity.

One influential factor in temporal construal is iconicity—a principle suggesting that the order in which events are mentioned in language also reflects their order in which they occur in the world. This principle is well-supported across studies in both discourse comprehension (Fleischman, 1990; Hopper, 1979; Horn, 2022; Kaiser, 2019; Kamp & Rohrer, 1983; Lascarides & Asher, 1991, 1993; Ohtsuka & Brewer, 1992; Schmerling, 1975; Tai, 1983; Xu & Kwok, 2019), and complex sentence comprehension (Blything & Cain, 2016; Blything et al., 2015; Clark, 1971; Clark & Clark, 1968; de Ruiter et al., 2018; Münte et al., 1998; Ohtsuka & Brewer, 1992; Trosborg, 1982; van der Meer et al., 2002; see also Dry, 2022).

In past-under-past relative clauses, iconicity seems to play a minor role. For instance, in Marx and Wittenberg (2024), participants read two sentences (e.g., *Mary got/was pregnant. She got/was married to John.*) which were crossed in dynamicity (i.e., eventive vs. stative) and in their linear order (i.e., pregnancy vs. marriage). Participants indicated which of the two situations had happened first. What determined temporal construal in this forced-choice task was dynamicity: States were consistently ordered before events. The same pattern was also found in relative clauses, using an act-out task (Marx et al., 2025) and sentence-video judgments (Marx & Wittenberg, 2022, 2025). Effects of iconicity were found, but they were second order, only relating to sentences that encoded two events. Furthermore, a systematic effect of iconicity in this condition across tasks was limited only to English (see Marx et al., 2025).

However, iconicity may play a larger role under certain conditions: If temporality is underspecified in future-under-future relative clauses, it may be underspecified overall, not just in terms of the temporal relation between the two described events. This might lead people to rely more on iconicity as a strategy to temporal construal. In this case, in a sentence in which the main clause precedes the relative clause, the event described in the main clause might be understood to precede the event described in the relative clause, irrespective of dynamicity differences between clauses.

#### Discourse-Pragmatic Function of Relative Clauses.

Another factor in temporal order construal may be the pragmatic function of certain linguistic constructions which serve to highlight or contrast new information with what is already known or part of the background (Chafe, 1970; Givón, 1983; Gundel, 1988; Hopper & Thompson, 1980; Kratzer, 2004; Krifka, 2008; Potts, 2004; Reinhart, 1981; Rizzi, 1997; Talmy, 1975; Tomlin, 1985). Relative clauses, in particular, have been described as syntactic tools that organize information pragmatically. Semantically, relative clauses either narrow the interpretative range of a noun phrase (i.e., restrictive relative clauses) or provide supplementary information about the referent (i.e., non-restrictive relative clauses, see Cinque, 2020; Fox & Thompson, 1990; Lambrecht, 1988; Levinson, 1983; Quirk et al., 1985; Stockwell et al., 1973; Thomson & Martinet, 1985).

Pragmatically, however, relative clauses often convey presupposed or given information, that is, details that the speaker assumes the listener is already familiar with or can readily infer. Especially within narratives, relative clauses have been associated with establishing the background of a story (cf. Chierchia & McConnell-Ginet, 1990; Depraetere, 1996; Ehrlich, 1987; Hopper, 1979; Potts, 2004; Reinhart, 1984; see also Ramsay, 1987; Talmy, 1975 for other subordinate clauses). By contrast, main clauses advance the narrative by detailing the temporal progression of events. Although the distinction between background and foreground information is not inherently temporal, it has been argued to bear a temporal dimension (Hopper, 1979; Hopper & Thompson, 1980; Polanyi Bowditch, 1976; Wårvik, 2004): Backgrounds need to be established before the foreground events occur.

Similar to iconicity, previous work demonstrates that comprehenders do not consistently adhere to the discourse pragmatic function of relative clauses—at least in past-under-past tense sentences that encoded both a dynamic event and a static state (Marx et al., 2025; Marx & Wittenberg, 2022, 2025). In such cases, dynamicity differences primarily guided temporal construal, with comprehenders showing little preference for backgrounding relative clause information. However, when dynamicity differences were absent, the pragmatic function of relative clauses did influence temporal interpretations, though this effect varied across languages, with English speakers displaying a weaker pattern (Marx et al., 2025).

It is therefore plausible that the discourse-pragmatic function of relative clauses might exert a stronger influence in future-under-future relative clauses, if the future primarily conveys modal meaning: In this case, the backgrounding nature of relative clauses could bias comprehenders toward interpreting the information conveyed by these clauses as temporally preceding the events described in the main clauses.

#### Figure-Ground Principle.

While the two alternative principles discussed so far—iconicity and the pragmatic function of relative clauses—offer plausible ways of interpreting relative clause constructions in the future, neither can fully account for previous findings in the past tense (Marx et al., 2025; Marx & Wittenberg, 2022, 2024, 2025).

However, there is a third possible explanation for the state-before-event inference: a general mechanism that structures representations into Figure and Ground in (nonlinguistic) cognition. This account would derive the state-before-event inference from dynamicity, independent of syntactic construction or tense.

Drawing on principles from Gestalt psychology, the Figure-Ground distinction originally described in perception highlights how a focal object (i.e., the Figure) at the center of attention is defined relative to a less focal or stable background (i.e., the Ground; Koffka, 1935). Conceptually applied to language, this distinction has been used to explain how syntax encodes spatial, temporal, and conceptual relationships (Gleitman et al., 1996; Levinson, 1996; Talmy, 1975; Zlatev, 2010): Specifically, the Figure is typically conceptualized as a “(conceptually) movable” entity that is defined relative to a more stable Ground entity (Gleitman et al., 1996, p. 358): In a sentence such as “The bike stood next to the house”, the bike, corresponding to the Figure, is more mobile, whereas the house, corresponding to the Ground, is both stable and large.

Gleitman et al. (1996) go on to say that the converse sentence “The house stood next to the bike” is odd precisely because some objects in relations to others make better Grounds, and others, better Figures. For instance, houses are large, and bikes are small. However, size alone does not determine what constitutes a better Figure or Ground, given that bikes are more prototypical Figures compared to other, smaller objects as well: “The bike stood next to the fire hydrant” sounds more natural than “the fire hydrant stood next to the bike”, despite the fire hydrant being smaller than the bike. This is, as we argue, and in line with Gleitman et al. (1996), because Grounds are ideally static, or at least relatively more static than Figures.

The conceptual structuring of scenes into agile Figure and static Ground can easily be extended into event cognition as well: States—being static and stable—are more prototypical Grounds, while events, characterized by motion or change, are more prototypical Figures. Thus, comprehenders may use a contrast in dynamicity to infer temporal relationships, independent of tense meaning. Interestingly, this view also aligns with conceptions in the discourse comprehension literature, which emphasize the role of dynamic properties in arranging information into situational foreground and background of a narrative (Hopper, 1979; Reinhart, 1984; Wårvik, 2004). Although these accounts use different terminology, they share the intuition that event dynamicity contributes to the organization of temporal and conceptual relations during language comprehension.^3^

A Figure-Ground account not only provides a coherent mechanism for temporal construal in future-under-future contexts, but also explains all previous findings for the past tense: In past-under-past relative clauses, people inferred that static states must precede dynamic events because states serve as a stable Ground for Figure events in the unfolding narrative. In future-under-future constructions, this mechanism should remain unchanged: Dynamic events should likewise be construed as Figures against the background of static states, so that they are reliably arranged after states in time. In both cases, the cognitive salience of events relative to states should drive the inference of temporal order, regardless of whether the temporal relation is located in the past or shifted into the future.

### The Current Studies and Predictions

The current studies were designed to test how wide the reach of the state-before-event inference is; and at the same time, as we argued above, our results should contribute to the debate on whether to conceive of the future as a tense, or as a modal.

The first, temporal anchoring account states that the state-before-event inference is derived from tense, based on the more salient event being an anchor for the less salient state. Therefore, it should predict that states precede events in the past tense, and states follow events in the future tense.

However, if the future tense is better thought of as a modal, we may expect effects driven by iconicity, whereby the first mentioned situation is always ordered first; effects driven by discourse-pragmatics in which relative clauses should be ordered first regardless of dynamicity; or, finally, effects driven by a Figure-Ground strategy, in which static states should precede dynamic events, regardless of order or syntax.

We conducted two preregistered experiments that used relative clause constructions to test the predictions derived from these two theoretical explanations as well as the formalizations of future tense in itself. Experiment 1: Past Tense served as a conceptual replication of prior findings on past-under-past relative clauses (Marx et al., 2025; Marx & Wittenberg 2022, 2025), employing an explicit temporal order judgment task (Marx & Wittenberg, 2024) to directly assess participants’ preferences of temporal order. This paradigm was chosen for two reasons: First, to ensure comparability with previous research, and second, due to its applicability in both past and future tense. In Experiment 2: Future Tense, we extended the design to future-under-future relative clauses to investigate the influence of tense meaning on temporal order construal. The results of these two experiments were compared in order to distinguish between the predictions outlined above (Results section).

## EXPERIMENT 1: PAST TENSE

### Participants

48 native English speakers were recruited through testable.com to participate in the experiment, which lasted approximately 10 minutes. We included only complete datasets in the statistical analysis; furthermore, datasets needed to meet our pre-registered attention criterion of at least 50% accuracy on filler trials. Based on these criteria, three participants were excluded, resulting in a final sample of 45 datasets for analysis.

### Materials

We constructed 16 critical sentence quadruplets similar to Marx and Wittenberg (2022, 2025) and Marx et al. (2025). All our stimuli were active, transitive and intransitive relative clause constructions with both predicates in the main and relative clause in the simple past.^4^ The event types of main clause (MC) and the relative clause (RC) were systematically manipulated throughout the four sentences: In (7), the main clause described a dynamic event while the relative clause described a static state; and in (8), the main clause described a state while the relative clause described a dynamic event. To investigate the role of alternative strategies of temporal construal in the absence of dynamicity differences in more detail, we included sentences like (9), where both the main and relative clause encoded stative descriptions, and sentences like (10), where both clauses encoded eventive descriptions.

(7)  eventive MC – stative RC
    *The tourist* [***took a picture***]_EVENT_ *of the monkey that* [***sat***]_STATE_ *in the tree.*
(8)  stative MC – eventive RC
    *The tourist* [***stood***]_STATE_ *next to the monkey that* [***swung***]_EVENT_ *in the tree.*
(9)  eventive MC – eventive RC
    *The tourist* [***took a picture***]_EVENT_ *of the monkey that* [***swung***]_EVENT_ *in the tree.*
(10)  stative MC – stative RC
    *The tourist* [***stood***]_STATE_ *next to the monkey that* [***sat***]_STATE_ *in the tree.*

In addition to the critical sentences, we created 24 complex filler sentences that explicitly encoded temporal order using various linguistic means: Sentences contained either temporal connectives (*before*, *after*), temporal adverbs (*and then*, *previously*), tense contrasts between the main and the relative clause (e.g., simple past vs. past perfect), or a combination of two of these strategies. In half of the filler sentences, the linear order of the sentence matched the encoded temporal order. In the other half, the temporal order was reversed, creating a mismatch between the temporal and linear order. All linguistic materials can be found at https://osf.io/sfdx9/.

### Procedure

In each trial, participants were presented with complex sentences such as those in examples (7–10) and were asked to make a temporal order judgment. Their task was to select one of two labeled buttons (e.g., *picture taking*, *standing*) to indicate which of the two described situations occurred first. The task prompt was *“What happened first?”* and the presentation side of the labels was randomized across trials to control for any potential side biases.

Participants completed a total of 41 trials: At the beginning of the experiment, participants completed a single practice trial to familiarize them with the procedure. Practice was followed by 16 critical trials (comprising 2 types of events in the main clause and 2 types in the relative clause, with 4 trials per condition) and 24 filler trials, all presented in a randomized order.

To counterbalance the conditions across subjects, participants were randomly assigned to one of four lists using a Latin-square design. Experiment 1 was programmed using PsychoPy and conducted online via Pavlovia.org.

### Statistical Analysis

We fitted a logistic mixed-effects regression model (i.e., *glmer* from the lme4 package; Bates et al., 2015) in the R statistics environment (R Core Team, 2014), including event type of the main clause (stative vs. eventive) and event type combination (mixed: eventive-stative/stative-eventive vs. same: eventive-eventive/stative-stative) as sum contrast-coded fixed effects, and participants and items as random intercepts to account for variability associated with these factors. As dependent variable, we coded whether participants chose the main clause to have happened first in time (main clause choices). We chose a minimal intercept-only structure with no random slopes to ensure model convergence and performed likelihood ratio tests to compare the full model with reduced models, excluding one of the predictors or the interaction at a time.

Furthermore, we conducted planned pairwise comparisons to further explore the interaction effects identified in the linear mixed-effects model. A preregistration of both experiments is available at https://osf.io/sfdx9/.

### Results

**Figure 3.**
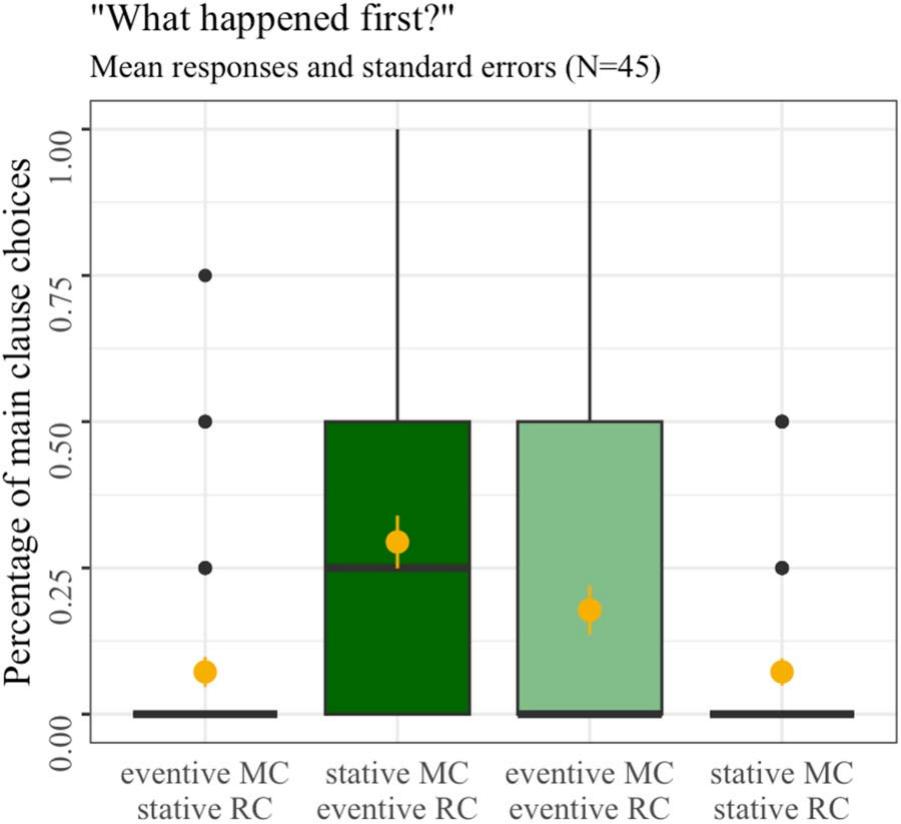
Mean main clause choices in Experiment 1 with boxplot summarizing means per participants in each condition. Yellow dots represent overall means, and yellow error bars the standard error of the mean.

Participants’ temporal judgments are displayed in Figure 3. Overall, we observed a general tendency for participants to order the relative clause first across conditions (*M*_OVERALL_ = 0.15, *SD* = 0.36). However, the strength of this tendency varied depending on the dynamic properties of the main and the relative clauses: For one thing, participants were more likely to judge the main clause as having happened first when both clauses encoded events (*M*_EVENT-EVENT_ = 0.18, *SD* = 0.38, light green third boxplot). More importantly, this pattern also held—and was even stronger—when the main clause encoded a state and the relative clause an event (*M*_STATE-EVENT_ = 0.29, *SD* = 0.46, dark green second boxplot).

The statistical analysis revealed no significant main effects of either event type of the main clause (*Df* = 1, *χ*^2^ = 3.07, *p* = 0.08) or event type combination (*Df* = 1, *χ*^2^ = 2.53, *p* = 0.11), but the interaction between both predictor variables turned out significant (*Df* = 1, *χ*^2^ = 41.76, *p* < 0.001): Stative main clauses were more often judged to have happened first when the relative clause encoded an event, compared to when the relative clause encoded a state as well (*M*_STATE-EVENT_ = 0.29, *SD* = 0.46 vs. *M*_STATE-STATE_ = 0.07, *SD* = 0.26; *β* = 0.11, *t* = −5.67, *p* < 0.001). At the same time, eventive main clauses were less often ordered first, according to participant’s temporal order judgments, when the relative clause encoded a state rather than when both clauses encoded an event (*M*_EVENT-STATE_ = 0.07, *SD* = 0.26 vs. *M*_EVENT-EVENT_ = 0.18, *SD* = 0.38; *β* = −0.05, *t* = −3.06, *p* = 0.002).

### Discussion

Our results replicate findings from previous studies using a temporal judgment design (Marx et al., 2025; Marx & Wittenberg, 2022, 2025): First, and foremost, the dynamic properties of the two situations described significantly influenced temporal construal in relative clause comprehension such that participants were more likely to order states before events.

However, participants also showed a strong tendency to interpret relative clauses as temporal backgrounds in all conditions—a pattern of results we predicted as a possible outcome for future-under-future, but not for past-under-past relative clauses based on the lack of it in previous studies. We suspect that the difference between Experiment 1 and previous findings may stem from the task design, which explicitly asked participants to reason about temporal relations within complex situations, rather than allowing temporal interpretation to emerge as an implicit by-product. This aspect, along with potential implications for experimental design in temporal comprehension, will be discussed in more detail in the General Discussion.

Finally, it should be noted that the relative-clause-first strategy was also weaker when both situations were framed as events—a finding that replicates a previously observed effect of iconicity, particularly for these conditions among English speakers (Marx et al., 2025; Marx & Wittenberg, 2024).

In Experiment 2, we investigated the influence of tense on temporal construal using future-under-future relative clause constructions. If tense meaning primarily determines the relationship between a situation and its temporal reference point, we predicted a reversal of the states-before-events effect, particularly in the mixed event-type conditions where the distinction between the temporal anchor and the anchored situation is clear-cut. Alternatively, if participants relied on a general Figure-Ground principle to construe temporal relations, we should find a replication of the pattern observed in Experiment 1. To assess the relative strength of these effects and to further clarify the role of future tense in temporal construal, we conducted a statistical comparison between Experiments 1 and 2.

## EXPERIMENT 2: FUTURE TENSE

### Participants

We recruited 48 native English speakers via testable.com, ensuring none had previously participated in Experiment 1. As in Experiment 1, we only included participants with complete datasets and accuracy rates above 50% on the filler trials. Four participants had to be excluded based on these criteria, resulting in 44 submitted datasets for analysis. Participation took approximately 10 minutes.

### Materials, Procedure, and Analysis

In Experiment 2, we used the same sentence structures as in Experiment 1, with the only modification being the change of tense to simple future across all critical sentences. The manipulation of event type in both the main and relative clauses remained the same as in Experiment 1 (see 11–14). For the filler trials, the only change made was the use of simple future tense. The same was true for the task prompt (i.e., *“What will happen first?”*).

(11)  eventive MC – stative RC
    *The tourist* [***will take a picture***]_EVENT_ *of the monkey that* [***will sit***]_STATE_ *in the tree.*
(12)  stative MC – eventive RC
    *The tourist* [***will stand***]_STATE_ *next to the monkey that* [***will swing***]_EVENT_ *in the tree.*
(13)  eventive MC – eventive RC
    *The tourist* [***will take a picture***]_EVENT_ *of the monkey that* [***will swing***]_EVENT_ *in the tree.*
(14)  stative MC – stative RC
    *The tourist* [***will stand***]_STATE_ *next to the monkey that* [***will sit***]_STATE_ *in the tree.*

The experimental procedure and statistical analysis in Experiment 2 were identical to those in Experiment 1 (i.e., mixed effect model with minimal intercept-only structure, planned pairwise comparisons). For the between-experiment comparison, we added tense as an additional predictor variable to the regression model to capture any difference in temporal construal between the simple past and the simple future tense.

### Results

**Figure 4.**
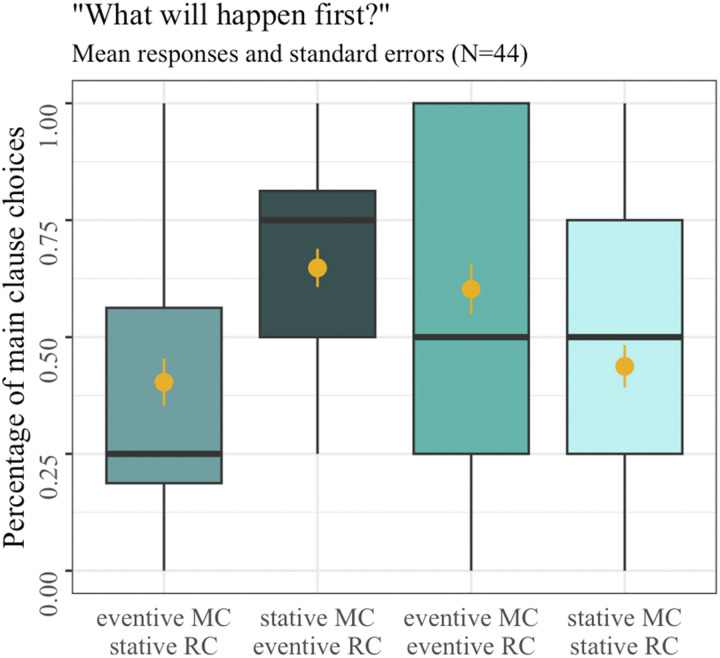
Mean main clause choices in Experiment 2 with boxplot summarizing means per participants in each condition. Yellow dots represent overall means, and yellow error bars the standard error of the mean.

The temporal judgments in Experiment 2, as shown in Figure 4, largely mirrored the patterns observed in Experiment 1. Participants were more likely to judge the main clause as happening first in two conditions: when both the main and the relative clause described an event (*M*_EVENT-EVENT_ = 0.60, *SD* = 0.49, teal third boxplot), and when the main clause described a state, and the relative clause described an event (*M*_STATE-EVENT_ = 0.65, *SD* = 0.48, slate second boxplot). This pattern was statistically supported by a significant interaction between the main clause event type and event type combination (*Df* = 1, *χ*^2^ = 45.86, *p* < 0.001), with pairwise comparisons revealing that: (a) stative main clauses were judged to occur first more often when combined with an eventive relative clause compared to a stative relative clause (*M*_STATE-EVENT_ = 0.65, *SD* = 0.48 vs. *M*_STATE-STATE_ = 0.44, *SD* = 0.50; *β* = 0.11, *t* = 4.04, *p* < 0.001); and (b) eventive main clauses were less likely to be judged as occurring first when combined with a stative relative clause compared to an eventive relative clause (*M*_EVENT-STATE_ = 0.40, *SD* = 0.49 vs. *M*_EVENT-EVENT_ = 0.60, *SD* = 0.49; *β* = −0.10, *t* = −3.80, *p* < 0.001). There were no main effects, neither for the event type in the main clause (*Df* = 1, *χ*^2^ = 2.15, *p* = 0.14) nor for event type combination (*Df* = 1, *χ*^2^ = 0.21, *p* = 0.65).

A notable difference between Experiments 1 and 2 was the absence of a relative clause-first strategy in Experiment 2 (*M*_OVERALL_ = 0.52, *SD* = 0.50). This was reflected by a significant main effect of tense in the cross-experimental analysis (*Df* = 1, *χ*^2^ = 58.73, *p* < 0.001). Additionally, a significant main effect of the event type in the main clause was observed (*Df* = 1, *χ*^2^ = 5.22, *p* = 0.02), along with a significant interaction between main clause event type and event type combination, consistent with the single-experiment results (*Df* = 1, *χ*^2^ = 81.33, *p* < 0.001). All other effects remained non-significant (i.e., main effect of event type combination: *Df* = 1, *χ*^2^ = 1.03, *p* = 0.31; interaction between main clause event type and tense: *Df* = 1, *χ*^2^ = 0.49, *p* = 0.49; interaction between event type combination and tense: *Df* = 1, *χ*^2^ = 1.72, *p* = 0.19).

### Discussion

Experiment 2 largely replicated the pattern of results from Experiment 1, with participants judging situations to happen first based on the dynamic properties of the two predicates: For future-under-future relative clause constructions, they were more likely to order static states before dynamic events, as predicted by only by a Figure-Ground account of temporal construal.

Similarly, we replicated the effect of iconicity from Experiment 1, in the absence of event dynamic differences: When both situations described events, participants tended to follow the linear order of the sentence to order situations in time.

In contrast to Experiment 1, we found no overall preference for locating relative clauses first in time: Choices between main and relative clauses were on average at chance level, with differences between conditions only due to differences in dynamicity encoding. While participants in Experiment 1 seemed to adhere to the discourse-pragmatic function of relative clauses as situational backgrounds—potentially influenced by the task design—participants in Experiment 2 did not incorporate this function into their temporal judgments. We propose that this difference between experiments supports the modal theory of the future: Since the individual temporal locations of each event as well as their relationship are underspecified, comprehenders have greater flexibility in determining temporal order. Interestingly, this flexibility appears to be mediated by a Figure-Ground principle and, in the absence of dynamicity differences, by iconicity.

## GENERAL DISCUSSION

The aim of this paper was to investigate the cognitive mechanisms underlying a robust phenomenon in the construal of temporally ambiguous language the states-before-events inference—and to test two alternative accounts. We asked: Does the states-before-events inference arise because people rely on temporal anchoring, using the temporal relationships encoded by grammatical tense? Or do comprehenders infer temporal order based on the dynamic properties of the described situations, following a Figure-Ground principle rooted in cognitive salience? In order to answer this question, we also explored questions about the meaning of the future tense: Is the future primarily a tense marker, encoding temporal relationships, or is it a modal marker, more connected to notions of future possibility?

In two experiments, we found a consistent pattern that not only replicated our previous findings regarding the states-before-events inference (Marx et al., 2025; Marx & Wittenberg, 2022, 2024, 2025) but also provided clear evidence for a Figure-Ground principle (Gleitman et al., 1996; Levinson, 1996; Talmy, 1975; Zlatev, 2010), extended to the event domain (see Hopper, 1979; Reinhart, 1984; Wårvik, 2004): Dynamicity influenced temporal order, regardless of any effect of syntactic encoding or tense. That is, dynamic events served as Figures against the Ground of static states, both when they were described as occurring in the past (Experiment 1) or in the future (Experiment 2).

Furthermore, in both experiments we found second-order effects of iconicity in cases where there were no differences in event dynamicity: Participants ordered events in time to match the reported order. Importantly, however, this pattern also demonstrates participants’ sensitivity to the difference between states and events: Only in sentences describing two eventive situations did iconicity shift temporal order construals to orders that followed the reported order. This replicates our previous findings (Marx et al., 2025; Marx & Wittenberg, 2024), but also suggests that, even in the absence of event dynamic differences, people use their knowledge about event structure and infer that events, rather than states, drive narratives forward in time (see Dry, 2022).

With regard to the states-before-events inference, one aspect of our data that requires further discussion is the observed preference for ordering relative clauses first in time in Experiment 1. This finding was unexpected, as it contradicts our initial hypotheses about past-under-past relative clause constructions and diverges from our previous findings using similar materials (see Marx et al., 2025; Marx & Wittenberg, 2022, 2025). Instead, it aligns with theories emphasizing the pragmatic function of relative clauses in discourse to establish situational (and temporal) background (Chierchia & McConnell-Ginet, 1990; Depraetere, 1996; Ehrlich, 1987; Hopper, 1979; Potts, 2004; Reinhart, 1984).

Why might this be the case? We suspect that this preference to order relative clauses first is driven by the design of the current study: In both experiments, participants were asked to reason about the temporal dimension of the sentences, providing explicit temporal order judgments. In contrast to previous methods (i.e., video-sentence matching or act-out tasks), which allowed for more implicit reasoning, the explicit focus on temporal order in the present study may have led participants to weigh linguistic and structural factors differently than in tasks that do not make temporal order the explicit question under discussion.

Similar shifts in processing strategies depending on how the question under discussion is framed have been observed in causal reasoning as well as in scope phenomena, presupposition and information structure, and other pragmatic inferences (e.g., Beaver et al., 2017; Gualmini et al., 2008; Marx & Wittenberg, 2022; Skordos & Barner, 2019). Even more broadly, the specifics of how linguistic experiments are designed may shift results in important ways. Various task characteristics—such as how stimuli are presented, how many fillers are used, how many response choices are available, or how they are labeled—can greatly affect the sensitivity of an experimental setup (Arehalli & Wittenberg, 2021; Marty et al., 2020; Schütze, 2016; Schütze & Sprouse, 2014).

However, unlike in these other studies, our main data pattern does not fundamentally change between tasks, populations, languages or, here, tense: There is a robust preference to order states before events. While the baseline preference may vary, we have consistently found people to draw this inference across all experiments we have ever run. As we have argued elsewhere, of course we would expect to find variance, and the effect should be overridden when stronger forces, such as causality, prevails, like in (15):

(15)  John planted persimmons that were delicious.

In order to be delicious, persimmons need to be planted first: When causality is strong enough, the states-before-events inference should be overridden (although perhaps being delicious could be attributed to any persimmons, past and future). The mechanisms and determining factors for such a possible disappearance of the states-before-events inference should be the topic of future research.

Another open question is how variation within the class of stative predicates may influence temporal construal (for discussion see also Marx et al., 2025; Marx & Wittenberg, 2025):^5^ In this study, we primarily examined stative descriptions of agents’ locations, relying mostly on posture verbs such as *to lean*, *to sit*, and *to stand*. However, stative predicates encompass a much broader range of meanings, including mental, physical, and possessive states, which could potentially affect how people infer temporal relations between described situations during language comprehension. While this possibility requires further investigation, initial evidence from Marx and Wittenberg (2024) suggests that this may not be the case: Testing a variety of stative predicates, we found a consistent pattern, indicating that the states-before-events-inference observed here extends beyond stative posture predicates.

Finally, our findings contribute to a long-standing debate in theoretical linguistics about theories of the future, which intersects with discussions in cognitive science about how people can mentally travel through time (Debus, 2014; Gautam et al., 2019; Redshaw, 2024; Suddendorf et al., 2009; Tulving, 1985). Both fields address the question of whether the future is simply another point on a timeline or refers to a non-deictic spectrum of possibilities. This distinction would also render reasoning about the future fundamentally different from reasoning about the past (Belnap et al., 2001; Perry, 2006). While other psycholinguistic research has investigated how people process the future (e.g., Biondo et al., 2022; Dickey, 2001), relatively little attention has been paid to temporal construal in complex constructions (but see Gennari, 2003; Dowty, 1982 for a theoretical discussion). The present study is a first step towards filling this gap.

## CONCLUSION

This study examined how comprehenders derive temporal order from ambiguous relative clause constructions, revealing a robust states-before-events inference as predicted by a Figure-Ground account of temporal construal. In two experiments, we show that salience differences between static states and dynamic events predict temporal construal across tenses in English, independent of tense meaning: Static states serve as the background for dynamic events. Importantly, our findings shed light on theories of future tense, suggesting that it is the modal nature of the future that introduces greater flexibility in temporal order judgments compared to the past. Overall, our results may advance our understanding of the origins of the states-before-events inference and illuminate how the interplay between tense, modality and event dynamicity shapes the construal of temporal relations from language.

## ACKNOWLEDGMENTS

We thank Ted Gibson and the two anonymous reviewers for their critical feedback, and the audience at AMLaP 2024 for their helpful comments.

## DATA AVAILABILITY STATEMENT

The data and analysis scripts are publicly available on OSF: https://osf.io/sfdx9/.

## Notes

^1^ To our knowledge, one of the few analyses of future-under-future sentences is Dowty’s (1982), whose conclusions align with the predictions we derive from the existing temporal anchoring theories. Dowty argues that a main clause future, functioning as a Priorian tense operator, shifts the reference time of any embedded tense. For example, in a sentence like *The tourist will take a picture of the monkey that will sit in the tree*, the relative clause takes the reference time of the main clause, situating it further into the future (see also Gennari, 2003).^2^ For our purposes, the term “underspecification” does not imply a strong commitment to a specific formal semantic theory. Nevertheless, we point out that this view of temporal order, based on the future as a modal, is highly compatible with possible world semantics (Heim & Kratzer, 1998; Partee, 1973). What matters here is that the ordering relations between two situations are underspecified in the future under a modal account.^3^ See also Sun et al. (2023), who demonstrate that the influence of the Figure-Ground distinction on temporality extends beyond the event domain to relations between event participants. In a picture-recognition task, participants more quickly matched linguistic descriptions to visual scenes when the Ground entity (e.g., a table) was displayed first, before the Figure entity (e.g., a vase on top of it), consistent with a Ground’s stable, background-like role.^4^ We exclusively used restrictive relative clauses, however, for a comparison with non-restrictive relative clauses, see Marx and Wittenberg (2025). Overall, the same pattern of results emerged for the two relative clause types, suggesting that this distinction does not critically impact temporal construal across these sentence constructions.^5^ We thank an anonymous reviewer for this suggestion.
